# Atrial myxoma with cerebellar signs: a case report

**DOI:** 10.1186/s13256-020-2356-5

**Published:** 2020-02-13

**Authors:** Suraj Shrestha, Akash Raut, Amar Jayswal, Randhir Sagar Yadav, Chandra Mani Poudel

**Affiliations:** Maharajgunj Medical Campus, Maharajgunj, Kathmandu, Nepal

**Keywords:** Cerebellum, Embolism, Myxoma, Stroke

## Abstract

**Background:**

Atrial myxoma remains a rare clinical entity with an incidence of surgically resected cases of 0.5–0.7 per million population and prevalence of < 5 per 10,000. It typically manifests in woman after third decade of life; symptoms vary greatly and may present with arrhythmia, intracardiac flow obstruction, embolic phenomenon, and associated constitutional symptoms. Neurological complications associated with atrial myxoma most frequently include cerebral infarct due to embolus. Cerebellar involvement is very rare and only a few cases have been reported in the literature.

**Case presentation:**

A 55-year-old Brahmin man with no history of diabetes mellitus and hypertension, presented with complaints of dizziness, headache, vomiting, double vision, and unsteadiness of gait for 2 weeks. His headache was sudden in onset, of a pulsating type and localized on left temporal side. Vomiting was projectile and bilious. Double vision was present in all directions of gaze and he had uncoordinated movement of his body and tilting to the left side. On examination, his cerebellar functions were impaired. He was thoroughly investigated for the cause of stroke after abnormal magnetic resonance imaging results with normal computed tomography angiography of his brain. Echocardiography and computed tomography of his chest showed a mass attached to intra-atrial septum and prolapsing through mitral valve, which was suggestive of left atrial myxoma. Five days following admission, he developed abdominal pain due to thromboembolism causing splenic and renal infarct.

**Conclusion:**

Although rare, atrial myxoma has to be considered a cause of stroke and other embolic phenomenon causing multiorgan infarctions. Early and timely diagnosis of the condition can prevent further recurrence and inappropriate anticoagulant therapy. It would be pertinent to have echocardiography done in patients who present with a stroke, arrhythmias, and other constitutional symptoms. The tumor once detected must be removed surgically as early as possible, which not only reduces serious thromboembolic complications but can be potentially curative.

## Background

Cardiac tumors are uncommon entities in medicine. Primary tumors of the heart are rare entities with an estimated incidence of less than 0.03% of which 75% are benign and half of them are myxomas. Cardiac myxoma (CM) still remains a rare clinical entity with an incidence of surgically resected cases of 0.5–0.7 per million population and prevalence of < 5 per 10,000 [1]. They generally occur between third and sixth decade of life and have a female preponderance. In 75% of cases the affected site is the left atrium and in 15–20% of cases the site is the right atrium. Embolic phenomenon occurs in 40–50% of cases [2].

Atrial myxomas are thought to originate from entrapped embryonic foregut. So, they are derived from multipotent mesenchymal cells capable of both neural and epithelial differentiation; hence, they tend to present with emboli. On microscopic examination, they contain scattered cells within a mucopolysaccharides stroma [3]. On gross examination they are pedunculated and gelatinous in consistency with a smooth, villous, or friable surface [4].

Clinical manifestations may vary greatly. In most cases, the tumor releases fragments or thrombi into systemic circulation leading to embolization and producing systemic and pulmonary manifestations. The most serious complication is neurologic, stroke being the commonest. Other manifestations include heart failure, arrhythmia, and pericardial effusion [4].

Currently, there is no effective medical treatment that arrests the growth of the tumor; thus, surgical excision of the tumor mass is the best modality of treatment. Here we report a case of a 55-year-old man who presented with complaints of headache, vomiting, double vision, and unsteady gait. He was extensively investigated to access the cause of the stroke and, finally, a rare diagnosis of atrial myxoma was made.

## Case report

A 55-year-old Brahmin man presented to our emergency department with complaints of headache, vomiting, double vision, and unsteady gait for 2 weeks. The sudden-onset headache, localized to left temporal region, was also associated with frequent bilious, non-bloody, projectile vomiting. He had reported double vision on the same day in all directions of gaze and unsteadiness of his lower limbs, which led him to tilt toward the left without support, and slowness and incoordination while walking. Furthermore, he had difficulty in holding objects and gripping. There was no fever, loss of consciousness, abnormal body movements, slurring of speech, nasal regurgitation, shortness of breath, chest pain, or palpitation. He occasionally consumed alcohol and was a tobacco smoker without any other significant medical or drug history and no family history of any cardiac disease.

On examination, he was alert, hemodynamically stable, and conversant. His vital signs were stable with blood pressure of 150/90 mmHg. Respiratory, cardiovascular, gastrointestinal, and thyroid examinations were otherwise unremarkable. A neurological examination revealed jerky nystagmus with fast component to the left. Hypotonia was present bilaterally on both upper and lower limbs. Past pointing was pronounced on his left hand with heel shin test affected on the left side. He had a wide-based gait and dysdiadochokinesia with normal speech findings suggestive of cerebellar involvement.

A complete blood count, lipid profile, thyroid function test, rheumatoid factor, and antinuclear antibody (ANA) test were normal. C-reactive protein (CRP) was positive and erythrocyte sedimentation rate (ESR) was increased to 47 mm/hour. Electrocardiography showed normal functioning heart. Magnetic resonance imaging (MRI) of his brain revealed acute/subacute posterior inferior cerebellar artery (PICA) territory infarct of the left cerebellum with small acute/subacute lacunar infarct of the left pons. Computed tomography (CT) angiography of cerebral vessels showed normal patency of vessels supplying his brain.

**Fig. 1:**
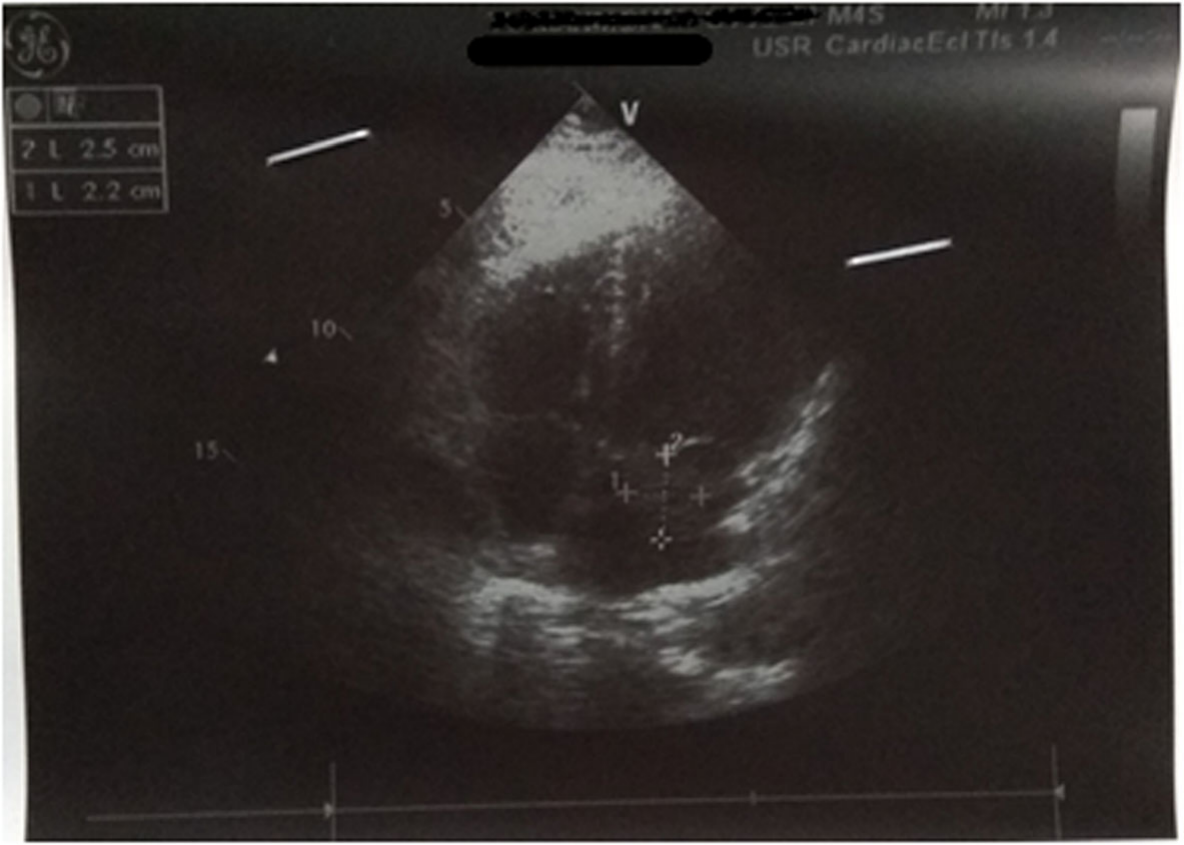
Mass attached to intra-arterial septum and prolapsing through the mitral valve. there were no clots/ vegetation and pericardial effusion

In order to assess the cause of infarct in his brain, transthoracic echocardiography was performed which showed a 2.5 cm × 2.2 cm mass attached to intra-atrial septum prolapsing through mitral valve with normal systolic and diastolic function suggestive of left atrial myxoma as shown in Fig. 1. Even though it is considered a surgical emergency, due to the financial and time constraints of our patient, it was planned that he would undergo surgery 6 weeks after presentation and follow-up every 2 weeks or whenever necessary. He was kept on anticoagulants.

**Fig. 2:**
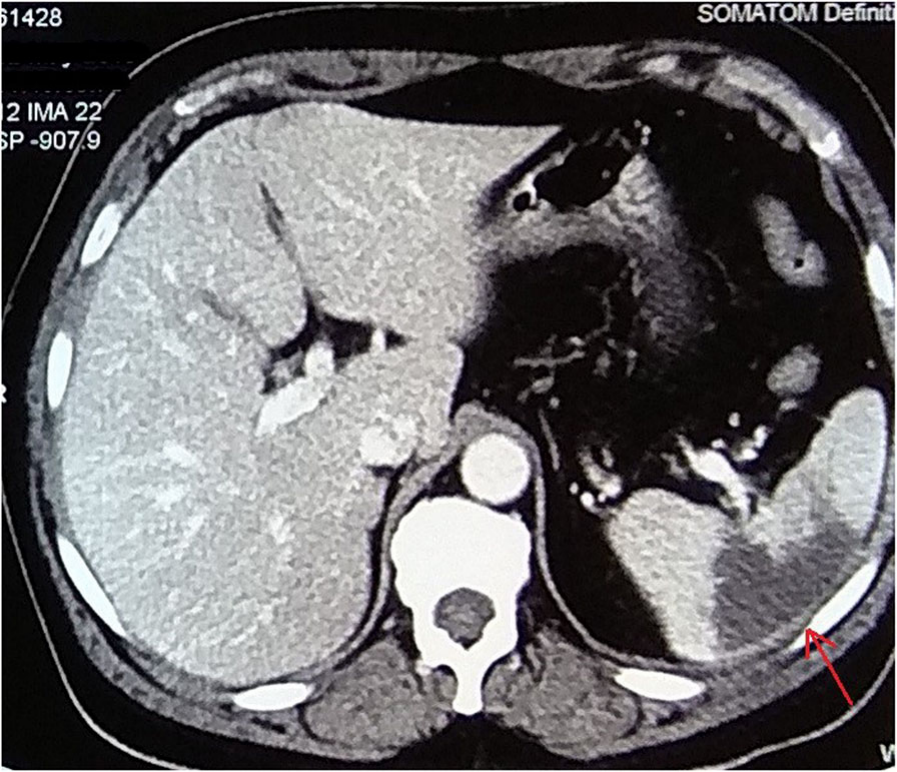
Hypodense lesion seen in the lower pole of spleen suggestive of splenic infarct

**Fig. 3:**
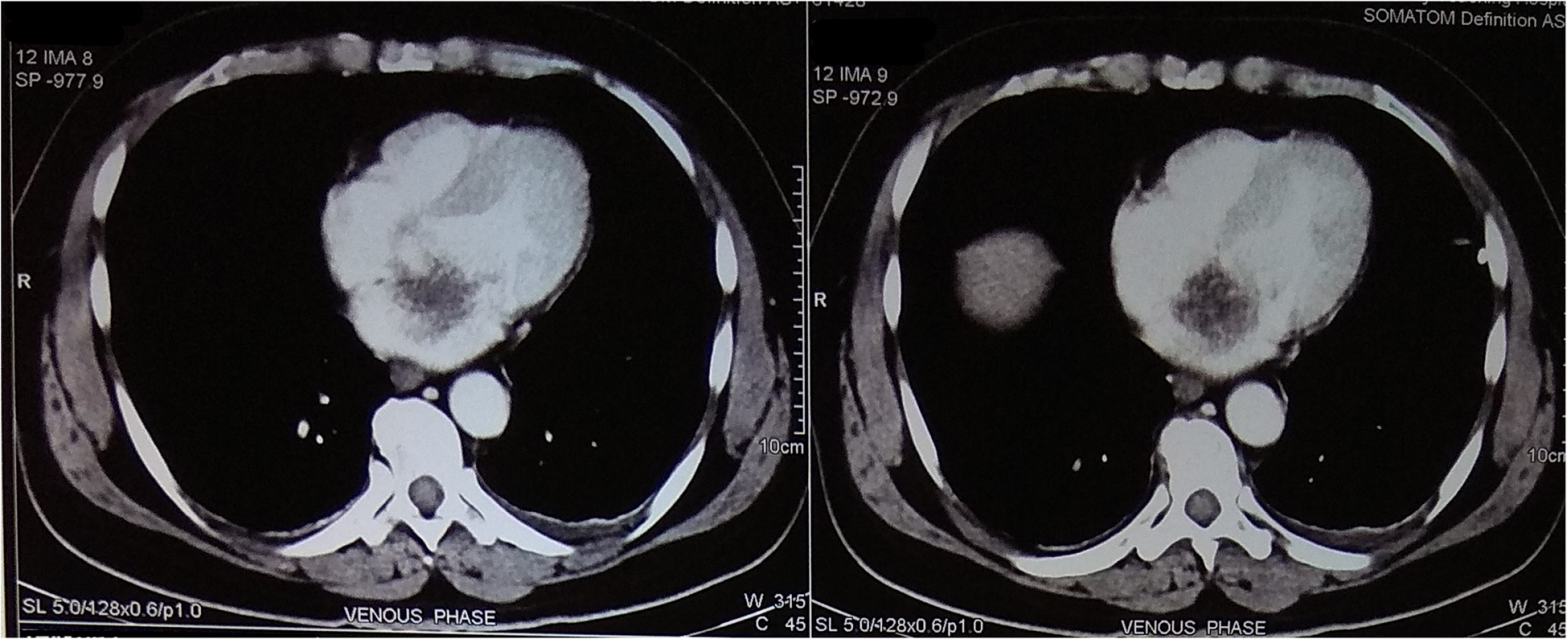
Irregular soft tissue density mass is seen in left atrium arising from interatrial septum

However, 5 days after admission, he complained of severe generalized abdominal pain for which a CT scan and other necessary investigations were advised. A CT scan of his abdomen and pelvis was performed which showed non-enhancing hypodense areas in lower pole of his spleen with a defect in one of the branches of the splenic artery suggestive of splenic infarct as shown in Fig. 2. Similarly, a wedge-shaped hypo-enhancing area was noted in bilateral kidneys which had extended into the sinus, which was suggestive of bilateral renal infarct. Furthermore, in a CT scan of his chest, an irregular non-calcified soft tissue density mass measuring 3.4 cm × 3.2 cm was seen in the left atrium arising from intra-arterial septum with non-enhancing areas suggestive of necrosis as shown in Fig. 3. All findings were evocative of left atrial myxoma.

**Fig. 4:**
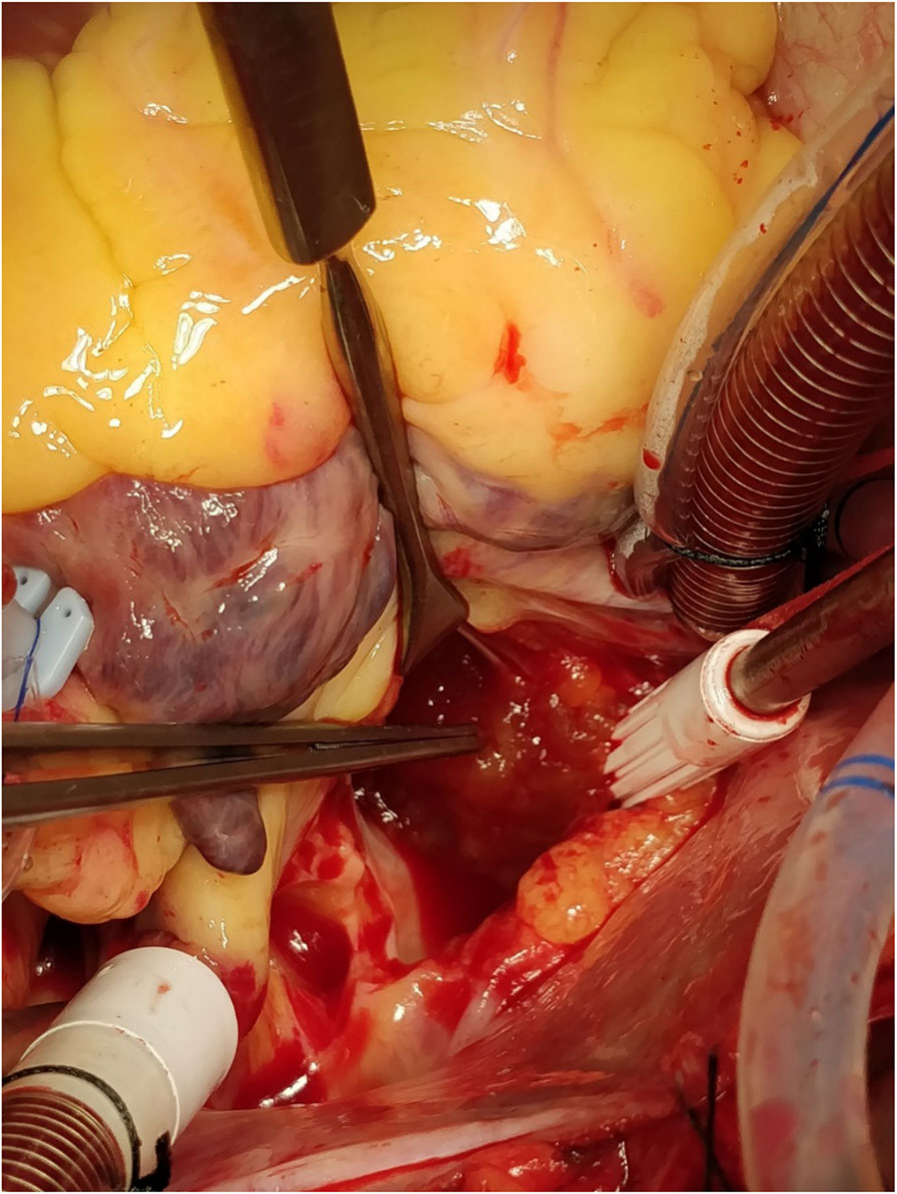
Operative intervention showing removal of tumor mass

He underwent excision of left atrial myxoma as shown in Fig. 4. His chest cavity was entered through median sternotomy. He was heparinized with checking of activated clotting time. Aorto-bicaval cannulation of 21 FR was done and a cardiopulmonary bypass (CPB) was established for 29 minutes. In order to minimize cardiac manipulation to prevent further embolic complications, an aortic cross-clamp was applied for 20 minutes. His heart was arrested with antegrade cold blood cardioplegia. Both the cavas were snugged and his right atrium was opened. The surgeon preferred development of Sondergaard’s groove to expose the left atrium. A 4 cm × 4 cm soft mulberry-shaped mass in our patient’s left atrium arising 1 cm away from posterior lateral commissure was excised. His left atrium was closed in layers and root was vented. His heart was de-aired and the aortic cross-clamp was removed. His heart did not require defibrillation and spontaneously returned to normal sinus rhythm. Upon reaching normothermia, he was weaned off from CPB on no pressor or inotropes. Protaminized decannulation was done and hemostasis was secured. His chest was closed in layers. He tolerated all the following procedures well and was monitored in an intensive care unit for 24 hours and moved to a ward later.

**Fig. 5:**
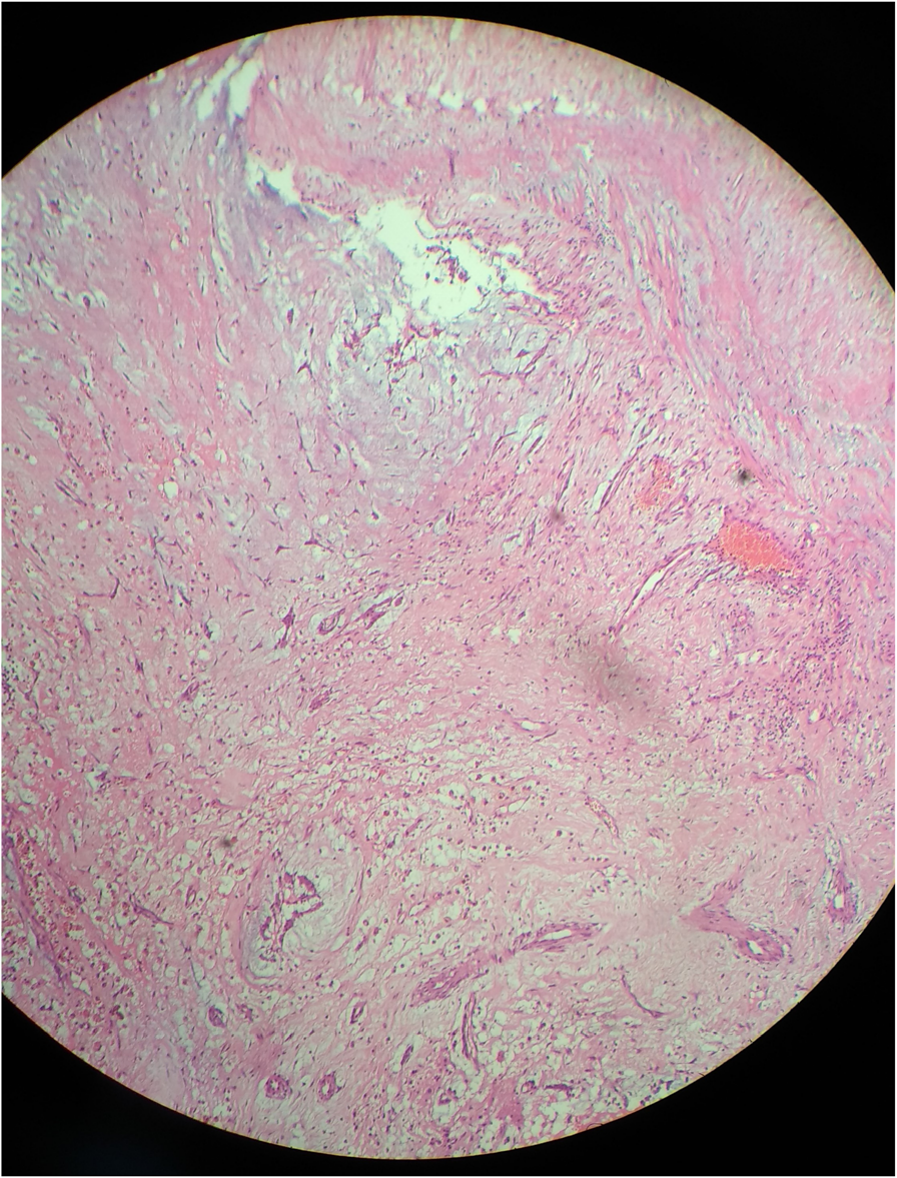
Excised mass showed marked loose myxoid stroma with scattered spindle cells without atypia confirming the diagnosis of atrial myxoma

Histopathological examination of the excised mass showed marked loose myxoid stroma with scattered spindle cells without atypia confirming the diagnosis of atrial myxoma as shown in Fig. 5.

His postoperative period was uneventful and he recovered well. He was doing well at 4-month post-surgery follow-up.

## Discussion

We reported a case of 55-year-old man with complaints of vomiting, headache, diplopia, and unsteadiness of gait for 2 weeks. The goals of the initial evaluation were to ascertain whether or not a stroke was present, the affected site of the brain, and, finally, the cause of the stroke.

Cerebellar stroke in itself contributes to only a small proportion of all strokes, commonly due to atherosclerosis and other vasculopathies. Atrial myxoma is an exceedingly rare cause of cerebellar stroke requiring a thorough investigation to reach to a diagnosis, which forms a basis for our discussion.

CM is the commonest of all primary cardiac tumors. The left atrium is the most common location for the myxoma, as in our case. Right atrial myxoma and ventricular myxomas are even rarer [5].

Clinical manifestations of atrial myxoma vary greatly and patients usually present one of the symptoms in the following tetrad: arrhythmias, intracardiac flow obstruction, embolic phenomena, and constitutional symptoms [6]. Embolic events occur in approximately 40–50% of patients with CM often to the brain due to fragments of the myxoma itself or surface emboli. In addition, embolization to the kidneys, spleen, aortic bifurcation, and the lower extremities is not infrequent [7]. Similarly, our patient had bilateral renal and splenic infarct due to tumor embolization along with the PICA territory infarct of left cerebellum and pons.

Stroke/transient ischemic attack (TIA) is the most common neurological presentation; most often in multiple sites with syncope, psychiatric symptoms, headache, and seizures [8]. Atrial myxomas that present with embolic phenomena causing stroke due to cerebral ischemia account for only 0.5% of all strokes [9]. In contrast to this, our case had cerebellar involvement which is a very uncommon presentation of atrial myxoma [10].

Patients with PICA territory infarcts most commonly present with acute vertigo, vomiting, headache, gait disturbances, and horizontal nystagmus ipsilateral to the lesion with headache being the most common initial symptom.

On clinical examination, left atrial myxoma may simulate many other diseases such as mitral regurgitation, pulmonary embolism, tricuspid stenosis, and tricuspid regurgitation [11]. No physical finding is pathognomonic. The “tumor plop” is often indistinguishable from an opening snap. Although a varying murmur is considered diagnostic, this is seen infrequently. Auscultation abnormalities may be absent in 36% of patients with myxoma, and a murmur suggestive of mitral stenosis has been reported in only 54% [12]. Our patient had no abnormal cardiac auscultatory findings as stated above.

Hemolytic anemia and thrombocytopenia, again often noted in left atrial myxomas, are thought to be due to the mechanical destruction of these blood elements by a mobile intraluminal tumor, which was absent in our case perhaps because of the size of the tumor [13].

Transthoracic echocardiography, a non-invasive method, is considered the imaging modality of choice for the diagnosis of CMs, but a transesophageal approach provides a better definition of the location and characteristics of the tumor with sensitivity of almost 100% [3]. Thus, a transesophageal echocardiogram (TEE) is preferred over transthoracic echocardiogram for evaluation of left atrial myxoma. As in our case, an intermittently prolapsing mass seen in echocardiography puts the patient at high risk of embolism [14].

MRI is more sensitive than CT in identifying subtle abnormalities of the brain as in stroke [15]. Moreover, MRI of the heart is especially useful in obese patients and in those patients with chronic obstructive airway disease, while echocardiography is less reliable [16]. Transthoracic and/or transesophageal echocardiography can be options for the screening of a stroke of a cardiac source [17].

In general, myxoma represents an emergency. Siminelakis *et al.* showed that urgent surgical resection via median sternotomy with the patient on CPB under mild hypothermia is the mainstay of treatment [18]. After diagnosis has been established, surgery should be performed as soon as possible as the risk of further tumor embolism and valve obstruction is too high while waiting for surgery, which is one of the dreadful complications [1, 8]. Considering the financial constraints of our patient, urgent surgery could not be performed which resulted in multiple infarcts a few days after the embolic stroke. Surgical resection is facilitated by utilizing cardioplegic arrest to provide a dry, motionless operative field to optimize complete excision and minimize perioperative tumor dislodgement and embolization, which was followed in our case [19]. However, the timing of surgery is controversial in patients who have had recent neurological insults and needs to be clarified as more experience is accrued [8].

Realistically, at stroke onset, one rarely knows if a patient has a myxoma, and it is difficult to evaluate all patients with echocardiography before starting thrombolysis because an incorrect diagnosis can lead to inappropriate treatment with anticoagulation as opposed to surgical resection [2]. Tumor resection is curative in most patients, and recurrent embolism after tumor removal is rare: recurring in 2–5% of sporadic cases and > 20% for cases of Carney complex. Hence, routine follow-up echocardiography is recommended [20, 21]. It seems that younger age, smaller size of the initial tumor, and tumor location in the ventricle were predictors of tumor recurrence [11]. The potential for malignant transformation is controversial, despite the publication of some reports of sarcomas arising from CM recurrences [22].

## Conclusions

CM often presents with an embolic phenomenon causing stroke. Cerebellum involvement is not common, which can easily mask myxoma as the differential diagnosis for the cause of stroke. A high degree of suspicion is necessary for early diagnosis and prevention of complications. The commonest means of reaching the diagnosis is echocardiography; it should be done in all cases of stroke as a screening tool to rule out any intracardiac mass, such as CM, as a source of emboli causing stroke. Urgent surgical excision of the mass remains the definitive treatment. Delay in immediate intervention can result in serious thromboembolic complications which can be life threatening. Regular long-term follow-up is necessary.

## Data Availability

All data are within the article.

## Abbreviations

ANA: Antinuclear antibody
CM: Cardiac myxoma
CPB: Cardiopulmonary bypass
CRP: C-reactive protein
CT: Computed tomography
ESR: Erythrocyte sedimentation rate
MRI: Magnetic resonance imaging
PICA: Posterior inferior cerebellar artery
TEE: Transesophageal echocardiogram
TIA: Transient ischemic attack
