# Correction to: Atrial myxoma with cerebellar signs: a case report

**DOI:** 10.1186/s13256-020-02364-2

**Published:** 2020-03-17

**Authors:** Suraj Shrestha, Akash Raut, Amar Jayswal, Randhir Sagar Yadav, Chandra Mani Poudel

**Affiliations:** Maharajgunj Medical Campus, Maharajgunj, Kathmandu, Nepal

**Correction to: J Med Case Rep (2020) 14:29**

**https://doi.org/10.1186/s13256-020-2356-5**

In the publication of this article [1], there is an error in the name of one of the contributing authors. This has now been updated in the original article.

The error:

Radhir Sagar Yadav

Should instead read:

Randhir Sagar Yadav
